# Supplementary material for: Elemental Screening and Nutritional Strategies of Gypsophile Flora in Sicily
Source: Plants (Basel). 2025 Mar 5;14(5):804. doi: 10.3390/plants14050804 (PMC11902630; doi:10.3390/plants14050804)
Supplement: Supplementary file 1 [file plants-14-00804-s001.zip › plants-3406967-supplementary.pdf]

Table S1

ANOVA results for element content among taxonomical families. Significant differences are indicated in bold.

Element	ANOVA	Sum of squares	df	Quadratic mean	F	p-value
C total (g/100g)	Between groups	277.352	3	92.451	13.332	0.000
	Inside groups	201.104	29	6.935		
	Total	478.456	32			
N total (g/100g)	Between groups	21.443	3	7.148	18.359	0.000
	Inside groups	11.290	29	0.389		
	Total	32.733	32			
Ca (g/100g)	Between groups	24.852	3	8.284	8.450	0.000
	Inside groups	28.432	29	0.980		
	Total	53.284	32			
S (g/100g)	Between groups	10.951	3	3.650	9.593	0.000
	Inside groups	11.035	29	0.381		
	Total	21.986	32			
Mg (g/100g)	Between groups	0.220	3	0.073	3.331	0.033
	Inside groups	0.637	29	0.022		
	Total	0.857	32			

Table S2

Summary of the multiple comparisons Scheffe Post Hoc tests performed for element content among taxonomical families. Significant differences are indicated in bold.

Element	Taxonomical family		Mean differences	Std D	p-value	95% confidence interval	
						Min	Max
C total (g/100g)	Asteraceae	Brassicaceae	10.270	1.756	0.000	5.061	15.479
		Cariophyllaceae	9.983	1.665	0.000	5.042	14.925
		Crassulaceae	7.925	1.862	0.003	2.401	13.449
	Brassicaceae	Cariophyllaceae	-0.287	1.110	0.995	-3.581	3.007
		Crassulaceae	-2.345	1.388	0.429	-6.463	1.773
	Cariophyllaceae	Crassulaceae	-2.058	1.272	0.466	-5.832	1.716
N total (g/100g)	Asteraceae	Brassicaceae	-0.228	0.416	0.959	-1.462	1.006
		Cariophyllaceae	-1.261	0.395	0.031	-2.431	-0.090
		Crassulaceae	0.876	0.441	0.288	-0.432	2.185
	Brassicaceae	Cariophyllaceae	-1.033	0.263	0.006	-1.813	-0.252
		Crassulaceae	1.104	0.329	0.021	0.129	2.080
	Cariophyllaceae	Crassulaceae	2.137	0.301	0.000	1.243	3.031
Ca (g/100g)	Asteraceae	Brassicaceae	-1.578	0.660	0.151	-3.537	0.380
		Cariophyllaceae	-2.873	0.626	0.001	-4.731	-1.015
		Crassulaceae	-2.337	0.700	0.022	-4.414	-0.260
	Brassicaceae	Cariophyllaceae	-1.295	0.417	0.038	-2.533	-0.056
		Crassulaceae	-0.759	0.522	0.557	-2.307	0.789
	Cariophyllaceae	Crassulaceae	0.536	0.478	0.741	-0.883	1.955
S (g/100g)	Asteraceae	Brassicaceae	-0.994	0.411	0.144	-2.214	0.226
		Cariophyllaceae	-1.367	0.390	0.015	-2.524	-0.209
		Crassulaceae	0.029	0.436	1.000	-1.265	1.323
	Brassicaceae	Cariophyllaceae	-0.373	0.260	0.569	-1.144	0.399
		Crassulaceae	1.023	0.325	0.034	0.058	1.988

	Cariophyllaceae	Crassulaceae	1.396	0.298	0.001	0.512	2.280
Mg (g/100g)	Asteraceae	Brassicaceae	-0.101	0.099	0.788	-0.395	0.192
		Cariophyllaceae	-0.212	0.094	0.190	-0.490	0.067
		Crassulaceae	-0.028	0.105	0.995	-0.340	0.283
	Brassicaceae	Cariophyllaceae	-0.110	0.063	0.392	-0.296	0.075
		Crassulaceae	0.073	0.078	0.832	-0.159	0.305
	Cariophyllaceae	Crassulaceae	0.183	0.072	0.112	-0.029	0.396

Table S3

Summary of ANOVA results for element content among functional types. Significant differences are indicated in bold.

Element	ANOVA	Sum of squares	df	Quadratic mean	F	p-value
C total (g/100g)	Between groups	102.606	2	51.303	4.095	0.027
	Inside groups	375.850	30	12.528		
	Total	478.456	32			
N total (g/100g)	Between groups	7.530	2	3.765	4.482	0.020
	Inside groups	25.203	30	0.840		
	Total	32.733	32			
Ca (g/100g)	Between groups	7.782	2	3.891	2.565	0.094
	Inside groups	45.502	30	1.517		
	Total	53.284	32			
S (g/100g)	Between groups	5.612	2	2.806	5.142	0.012
	Inside groups	16.374	30	0.546		
	Total	21.986	32			
Mg (g/100g)	Between groups	0.006	2	0.003	0.108	0.898
	Inside groups	0.851	30	0.028		
	Total	0.857	32			

Table S4

Summary of the multiple comparisons Scheffe Post Hoc tests performed for element contents among functional types. Significant differences are indicated in bold.

Element	Functional type		Mean differences	Std D	p-value	95% confidence interval	
						Min	Max
C total (g/100g)	Narrow gypsophile	Gypsovag	3.928	1.445	0.037	0.207	7.650
		Wide gypsophile	4.047	1.866	0.112	-0.757	8.851
	Wide gypsophile	Gypsovag	-0.119	1.669	0.997	-4.416	4.178
N total (g/100g)	Narrow gypsophile	Gypsovag	-0.870	0.374	0.083	-1.834	0.093
		Wide gypsophile	0.200	0.483	0.918	-1.044	1.444
	Wide gypsophile	Gypsovag	-1.070	0.432	0.061	-2.183	0.043
Ca (g/100g)	Narrow gypsophile	Gypsovag	-1.126	0.503	0.098	-2.421	0.169
		Wide gypsophile	-0.939	0.649	0.364	-2.610	0.733
	Wide gypsophile	Gypsovag	-0.188	0.581	0.949	-1.683	1.307
S (g/100g)	Narrow gypsophile	Gypsovag	-0.962	0.302	0.013	-1.739	-0.185
		Wide gypsophile	-0.753	0.389	0.172	-1.756	0.250
	Wide gypsophile	Gypsovag	-0.209	0.348	0.836	-1.106	0.688
Mg (g/100g)	Narrow gypsophile	Gypsovag	0.026	0.069	0.932	-0.151	0.203
		Wide gypsophile	0.038	0.089	0.912	-0.191	0.267
	Wide gypsophile	Gypsovag	-0.012	0.079	0.988	-0.217	0.192

Table S5

Summary of the T-test results for element contents for succulence characters. Significant differences are indicated in bold.

Element	T-Test	Sum of squares	df	Quadratic mean	F	p-value
C total (g/100g)	Between groups	62.384	1	62.384	4.648	0.039
	Inside groups	416.072	31	13.422		
	Total	478.456	32			
N total (g/100g)	Between groups	0.536	1	0.536	0.516	0.478
	Inside groups	32.197	31	1.039		
	Total	32.733	32			
Ca (g/100g)	Between groups	8.067	1	8.067	5.531	0.025
	Inside groups	45.217	31	1.459		
	Total	53.284	32			
S (g/100g)	Between groups	2.860	1	2.860	4.636	0.039
	Inside groups	19.126	31	0.617		
	Total	21.986	32			
Mg (g/100g)	Between groups	0.009	1	0.009	0.316	0.578
	Inside groups	0.848	31	0.027		
	Total	0.857	32			
